# Climate Change‐Driven Heatwaves Pose Lethal Risks to Newborn Forest Bats

**DOI:** 10.1002/ece3.71350

**Published:** 2025-05-13

**Authors:** Danilo Russo, Anne Mäenurm, Luca Cistrone, Adriano Martinoli, Greta Foiani, Valentina Giongo, Stefania Leopardi

**Affiliations:** ^1^ Laboratory of Animal Ecology and Evolution (AnEcoEvo), Dipartimento di Agraria Università degli Studi di Napoli Federico II Napoli Italy; ^2^ AFNI Friuli‐Venezia Giulia Cordenons Italy; ^3^ Unità di Analisi e Gestione delle Risorse Ambientali, Guido Tosi Research Group, Dipartimento di Scienze Teoriche Ed Applicate Università degli Studi dell'Insubria Varese Italy; ^4^ Istituto Zooprofilattico Sperimentale Delle Venezie Legnaro Italy

**Keywords:** bats, climate change, ecological trap, forest, heatwaves, mortality

## Abstract

Climate change poses a significant threat to biodiversity, with extreme weather events such as heatwaves exacerbating the risks to animal populations. Temperature extremes can cause high physiological stress in animals, particularly in species or life stages with limited thermoregulatory abilities. While available evidence pertains to flying foxes and bats using bat boxes or dwelling in urban environments, heatwave‐induced mortality in forest‐dwelling species in temperate forests has not been reported. We present the first evidence of heatwave‐related mortality in temperate forest bats, specifically in common noctules *Nyctalus noctula* , observed in northeast Italy during the summers of 2023 and 2024. Our fieldwork, conducted in a forest fragment in the Friuli‐Venezia Giulia Region (Northeastern Italy), identified 17 dead juvenile bats found at the base of roost trees during periods of extreme heat (T_max_ ≥ 30°C). Laboratory necropsies revealed that the cause of death was consistent with heat‐related stress, as no viral infections were detected, and recent feeding evidence was present in a few individuals. Dead bats are difficult to find in forests, especially when mortality occurs in unsurveyed areas, scavengers remove carcasses, or deaths go unnoticed within roost cavities. Consequently, our observations likely represent only a limited fraction of actual mortality. The phenomenon may be quantitatively significant and widespread. The findings highlight the vulnerability of bat populations to heatwaves, particularly in fragmented forest habitats where roosting opportunities are limited. Our results allow us to hypothesise that forest fragmentation increases exposure to heat stress, particularly along forest edges. In the context of climate change, roosts deemed suitable may act as ecological traps, making this a hypothesis worth testing.

## Graphical abstract

This is the first recorded case of forest bat mortality in temperate regions caused by heatwaves. The study suggests that forest fragmentation exposes bats to lethal thermal peaks, highlighting a previously overlooked impact of climate change on these mammals and potential interactions with forest fragmentation.

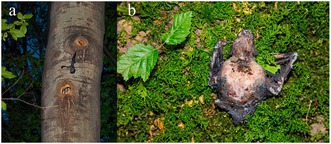

## 1 Introduction

Climate change is one of the most pressing threats to biodiversity (Schlaepfer and Lawler 2023). Long‐term shifts in temperature and precipitation patterns, alongside an increased frequency of extreme weather events such as heatwaves, profoundly affect animal physiology and behaviour, with adverse consequences for biodiversity and ecosystem functions (Maibach et al. 2019). Among the most immediate effects of climate change are temperature extremes, which, when intense and prolonged, impose physiological stress across species (Wang et al. 2024).

Rising temperatures and extreme events can directly and indirectly impact animal survival, reproduction and behaviour. Species with limited thermoregulatory abilities are particularly vulnerable, facing physiological stress, reduced habitat suitability and altered ecological interactions (Stillman 2019). These effects are especially pronounced in endotherms, which employ significant energy in maintaining stable internal temperatures (Fuller et al. 2014). Heat stress during critical life stages such as reproduction, development, and dispersal can lead to reduced offspring survival, population declines, and disruptions to community structure (Stillman 2019).

Bats, one of the most ecologically diverse and widely distributed mammalian groups (Simmons and Cirranello 2024), are highly susceptible to climate‐induced environmental changes (Festa et al. 2023). While much of what is known about bats' responses to climate change comes from model predictions (e.g., Smeraldo et al. 2021), growing evidence suggests that they are already reacting through shifts in phenology (Haest et al. 2021), geographic range (Ancillotto et al. 2016) and body mass (e.g., Mundinger et al. 2023; Russo, Jones, Polizzi, et al. 2024; Russo, Jones, Martinoli, et al. 2024). Bats are sensitive to heatwaves, as their large surface‐area‐to‐volume ratio increases susceptibility to acute dehydration on hot days (Korine et al. 2016). This sensitivity has led to mass mortality events in flying foxes (Ratnayake et al. 2019) and insectivorous bats roosting in buildings (Pruvot et al. 2019) and bat boxes (Crawford and O'Keefe 2021). Reproductive females benefit from warmer roost microclimates, which reduce the energetic costs of maintaining homeothermy during pregnancy and lactation (Gittleman and Thompson 1988). However, attraction to warmer sites also increases their vulnerability to heatwaves, which can turn seemingly suitable roosts into ecological traps (Salinas‐Ramos et al. 2023).

Small bats roosting in buildings or bat boxes are vulnerable to heatwaves. Confined to narrow spaces with limited movement, such bats are highly susceptible to sudden temperature spikes (Salinas‐Ramos et al. 2023). While heatwave‐induced mortality also affects adult bats, newborns are particularly at risk due to their limited thermoregulatory capacity and mobility (Sano 2000; Sun et al. 2020). Heatwave‐induced mortality may seem less likely for bats roosting in temperate forests, as tree cavities are generally more abundant than other roost types, and forest bats frequently switch roosts (Willis and Brigham 2004; Russo et al. 2005, 2017; Reckardt and Kerth 2007). In principle, unlike bats confined to rarer roost structures, such as suitable spaces in buildings, tree‐roosting bats may abandon overheated roosts in favour of cooler sites, thus mitigating the effects of heatwaves.

Managed forests provide progressively fewer roosting opportunities, as intensive silviculture practices promote the removal of veteran or decaying trees that offer essential cavities (Wainhouse and Boddy 2022). Reduced roost availability may force bats to use suboptimal sites and diminish roost‐switching options, while forest thinning increases sun exposure by reducing canopy cover (Hale 2003). Fragmentation further exacerbates these effects by exposing trees at forest edges to direct sunlight (Mann et al. 2023). Consequently, human‐driven forest alterations, in combination with climate change, are expected to impact tree‐roosting bats.

Die‐offs related to heat stress have been well documented in flying foxes, which form large colonies hanging from trees (Mo et al. 2021). However, mortality in insectivorous bats inhabiting temperate forests is less readily observable and difficult to quantify due to limited human presence, the bats' small size, and frequent scavenging. These challenges also make it difficult to detect clinical signs of sub‐lethal heat stress or preceding death and result in the rapid decomposition of carcasses, often limiting diagnostic opportunities. While other factors, such as infectious agents or chemical pollutants, could contribute to similar die‐offs (O'Shea et al. 2016), identifying the primary cause remains challenging. Here, we provide the first evidence supporting heatwave‐induced newborn mortality in temperate forest bats, following extreme heat events in Northeastern Italy in June 2023 and 2024. To refine our hypotheses regarding the cause of death, we conducted necroscopy and microbiological analyses on retrieved carcasses, allowing us to assess potential alternative explanations and strengthen the link to heat stress.

## 2 Material and Methods

### 2.1 Field Observations

**FIGURE 1:**
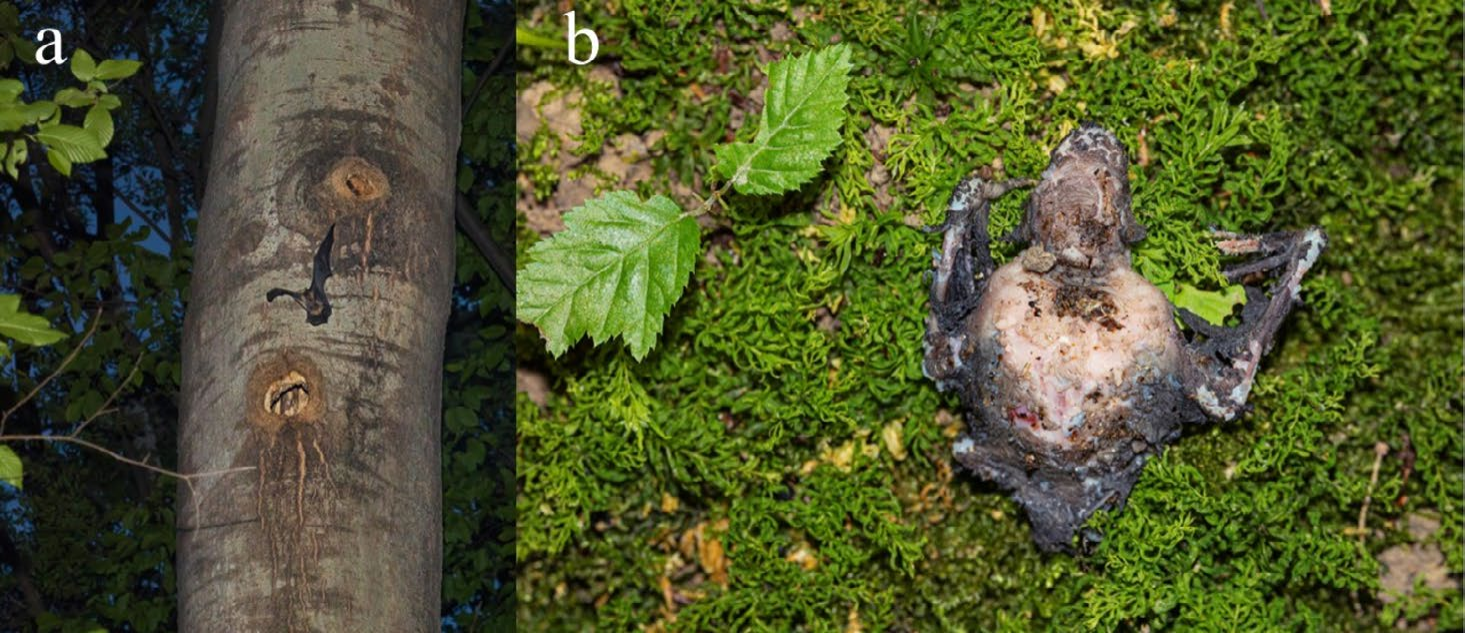
Typical noctule roost in the study area (a) and a dead newborn found at the base of a roost tree in June 2024 (b).

Our observations were part of an ongoing monitoring initiative of *Nyctalus lasiopterus* and *Nyctalus noctula* conducted since April 2021 in the Friuli‐Venezia Giulia Region near Udine (46.0711° N, 13.2346° E) by A.M. The study site consists of a 20‐ha forest fragment at 15 m a.s.l. where *N. lasiopterus* and *N. noctula* roost year‐round (Russo et al. 2023) located at ca. 15 m above sea level. The site is surrounded by agricultural fields, primarily corn, wheat, and vineyards, and features several tree species, including European aspen, European hornbeam, and pedunculate oak. The fragment is one of the few remains of the once‐widespread “Silva lupanica” forest (Mäenurm, 2021). The bats select roosts based on specific tree characteristics, favouring aspen and other cavity‐rich trees. They prefer sites with high canopy closure in the forest interior and frequently use decay‐formed cavities (Figure 1; Russo et al. 2023; Russo, Mäenurm, Martinoli, et al. 2024).

Roost monitoring was conducted three to four times per week from March to November, with surveys intensifying daily in June and July. Roost locations were identified using multiple approaches, including recognising the characteristic social calls of *Nyctalus* colonies before sunset, photographing bats within cavities during the day, and observing their emergence at dusk. We focused on 20 roost trees previously identified as occupied by bats, including four key maternity roosts. Surveys were conducted systematically, ensuring that each identified roost was inspected thoroughly. Additional details are available in Russo et al. (2023). In June 2024, after the first newborn of the season was found dead at the base of a roost tree, survey efforts were intensified. We inspected the area surrounding each tree daily in the early morning to minimise the risk of bats being scavenged by red foxes and other small carnivores. During these intensified surveys, we checked the primary maternity roosts and other potential roost trees to establish if mortality occurred. No dead adult bats were found, nor was there any evidence of newborn mortality under normal temperature conditions. Additionally, no other dead wildlife was observed in the surveyed area during this period.

Dead bats were collected immediately, placed in sterile containers, and deep‐frozen at −18°C. Samples were kept at a controlled temperature during transportation to the Istituto Zooprofilattico Sperimentale delle Venezie (IZSVe) using insulated containers. Laboratory analyses to establish the cause of death were then conducted upon arrival.

### 2.2 Laboratory Analyses

Species identification of noctule bats was performed through DNA barcoding using the cytochrome c oxidase subunit I (COI) gene. PCR amplification and sequencing were performed using brain tissue (de Benedictis et al. 2022), and the resulting sequences were compared against reference databases (GenBank, BOLD) for species‐level identification.

A necropsy was performed on intact carcasses, examining organs for the presence of gross lesions. We inspected the whole length of the gastrointestinal tract, the heart, and the liver to investigate the presence of adult parasites. We collected tissue samples for histological evaluation from the liver, intestine, spleen, kidney, lung, heart, brain and hind limb, including skeletal muscle, tibia and associated joints. Tissue samples were fixed in 10% neutral buffered formalin and processed with haematoxylin and eosin (H&E) staining. Hind limb samples were decalcified after fixation for 1 h at room temperature using Leica Decalcifier I (Leica Biosystems, Buccinasco, Milan, Italy).

To investigate viral infections as a potential cause of death, we screened organ pools from suitable carcasses for the presence of viruses known to circulate in bats in the area and potentially associated with mortality, using broad‐spectrum molecular tests. Organs were homogenised in lysis buffer using the TissueLyser (Qiagen, Hilden, Germany), and genetic material was extracted with the NucleoSpin RNA mini kit (Macherey‐Nagel) following the manufacturer's instructions. The presence of Lyssavirus RNA in brain samples was tested using a one‐step real‐time RT‐PCR (rRT‐PCR) with AgPath‐ID One‐Step RT‐PCR Reagents (Thermo Fisher, Waltham, USA), as described by Drzewnioková et al. (2023). All other viruses were tested from pooled samples containing small portions of all available organs to maximise screening sensitivity. Molecular tests included nested RT‐PCRs for Coronaviridae and *Orthohantavirus*, performed as previously described (Drzewnioková et al. 2021; Leopardi et al. 2022). Additionally, Filoviridae screening was conducted with a nested RT‐PCR using OneStep RT‐PCR (Qiagen) and Platinum Taq (Invitrogen) for the two amplification steps, along with a primer mix from the literature (Zhai et al. 2007).

### 2.3 Statistical Analysis

Daily observations of maximum temperature (T max) were taken from 3BMeteo, an online meteorological service (www.3Bmeteo.com). Because the exact time of death could not be determined precisely, we aggregated the data into three‐day intervals to reduce the noise associated with daily fluctuations. For each interval, we calculated the mean T max to represent the average environmental conditions and summed the counts of dead bats to capture the mortality events throughout that period. This aggregation provided a more stable basis for analysis and minimised potential misclassification of the day of death. The count data exhibited overdispersion, so we employed a Generalised Linear Model with a negative binomial distribution and a log link function, using the number of dead bats as the dependent variable and mean maximum temperatures over 3‐day intervals as the predictor. We limited the analysis to 2024 data since the 2023 sample size was insufficient.

## 3 Results

**FIGURE 2:**
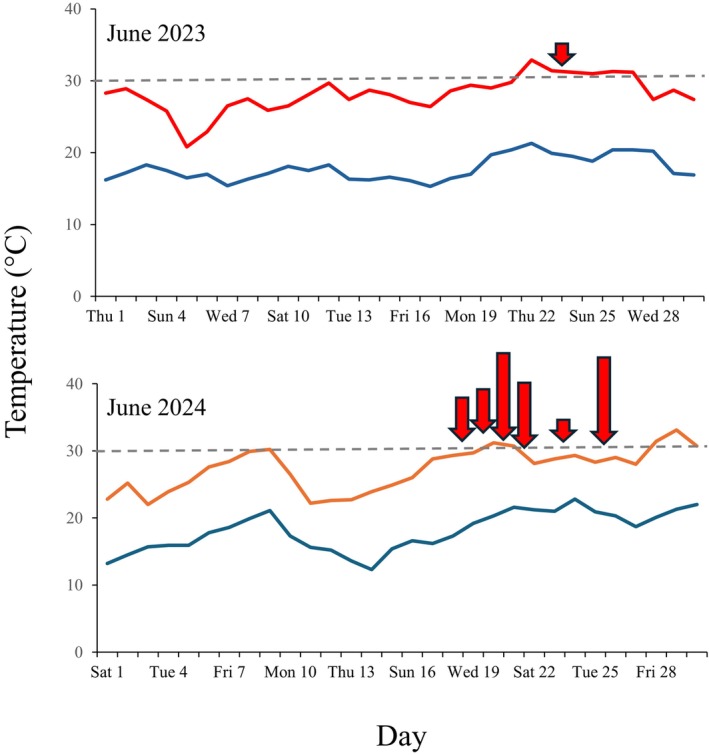
Maximum (red line) and minimum (blue line) temperatures in Cervignano del Friuli, Udine (NE Italy) in June 2023 and 2024. The arrows indicate the dates when dead bat pups were retrieved, with arrow length proportional to the number of bats recovered (range 1–4 per day). The woodland patch was surveyed during the entire period covered in the graph.

We collected 17 dead bats, one in June 2023 and the others in June 2024, all at the base of three known roost trees (Figure 1). Their state of preservation varied depending on the time since death and local conditions at the retrieval site, such as insulation and the amount of leaf litter (Table S1). In all cases, the discovery of dead bats was associated with ambient temperatures approaching or exceeding 30°C (Figure 2). The GLM statistically confirmed this pattern, showing a significant log‐likelihood ratio test for T max (*χ* ^2^ = 11.5, df = 1, *p* < 0.001) and AIC = 51.9 vs. a null model's AIC = 61.4. The roost trees were all relatively close to the forest patch edge (39, 81 and 51 m; the maximum distance from the forest patch centroid to its edge was 424 m.).

Molecular species identification was possible for 10 bats, all classified as common noctules ( *Nyctalus noctula* ). Results of necropsy, histology and virological screening are summarised in Table S1. All individuals were neonates or only a few days old, with one showing incomplete skull ossification. Sex could be visually assessed for four females and three males. Six individuals were suitable for necropsy and exhibited a moderate to advanced state of decomposition, particularly affecting the liver, intestines and lungs. No relevant gross lesions were observed, except in one individual with congested lungs with areas of pallor. Notably, this bat showed evidence of recent feeding, as indicated by milk in the stomach. Similarly, faeces were present in the rectum of another bat. Histological examination confirmed advanced post‐mortem autolysis and bacterial overgrowth. We detected no significant histopathological lesions despite poor tissue preservation. In lung (*n* = 3) and liver (*n* = 2) samples with better tissue preservation, moderate to high numbers of polymorphonuclear and mononuclear intravascular cells—consistent with neutrophils, eosinophils, immature leucocytes, and haematopoietic cells—were detected. These cells were occasionally observed in the lung alveolar spaces, along with sparse activated macrophages. No adult parasites were detected in the gastrointestinal tract, heart or liver of the bats. Tested individuals scored negative for lyssaviruses (*n* = 8), coronaviruses (*n* = 6), hantaviruses (*n* = 6) and filoviruses (*n* = 6).

## 4 Discussion

We present the first documented case of tree‐dwelling bat pups killed by heatwaves in temperate regions, with necropsy and virological analysis excluding other major potential causes of mortality. This observation builds on previous research showing that bats roosting in sun‐exposed artificial structures, such as bat boxes and buildings (e.g., Salinas‐Ramos et al. 2023), are at risk of heat‐related mortality, especially during heatwaves. Artificial roosts may overheat on hot days (Martin Bideguren et al. 2019; Crawford and O'Keefe 2021; Czenze et al. 2022b; Lourenço and Palmeirim 2004), causing bat dehydration and heat stress (Alcalde et al. 2017; Flaquer et al. 2014). Heatwaves' frequency and intensity are predicted to increase as climate change exacerbates (IPCC 2021), placing additional pressure on bat populations. Moreover, heatwaves are expected to occur earlier in the year (IPCC 2021), which could increase the risks to bats. All the bat carcasses available for examination belonged to unweaned individuals. In most cases, mass die‐offs of pups are attributed to starvation and dehydration following abandonment by nursing females (Mo et al. 2021). However, in our case, at least two of the six necropsied individuals had recently been fed, as evidenced by milk in the stomach and faeces in the intestines. This suggests that factors other than maternal abandonment, such as environmental stressors, likely contributed to their mortality.

Heat stress is typically diagnosed through clinical evaluation and pathological examination (Black and Mitchell 2009). In our case, all individuals were found dead, limiting our assessment to post‐mortem findings. Heat stress in humans and domestic animals is often associated with multi‐organ oedema, congestion, haemorrhages, and coagulative necrosis, with key affected organs including the brain, kidney, liver, heart, and skeletal muscle (Black and Mitchell 2009; Bruchim et al. 2009). These lesions are linked to endotoxaemia and coagulation cascade activation following severe hyperthermia (Bruchim et al. 2009). In our study, necropsied bats did not show signs of haemorrhages. However, our ability to detect heat‐related lesions was constrained by the small sample size and the moderate to advanced decomposition of the carcasses. The severe post‐mortem tissue changes, likely resulting from the rapid onset of autolysis and bacterial overgrowth under high ambient and body temperatures, significantly hindered histological analysis. Interestingly, we observed an accumulation of intravascular leucocytes in some cases, which may reflect leukocyte activation in response to physiological stress or inflammation (Xie et al. 2019). Although this finding is nonspecific, similar intravascular accumulation of neutrophils in the liver has been reported in a baboon model of heat stroke (Bouchama et al. 2005).

The pathophysiology of heat‐related mortality in bats may differ from that in other mammals. Several bat species exhibit unique immune adaptations that modulate inflammatory responses and limit tissue damage (Baker et al. 2013; Banerjee et al. 2020). Additionally, microbats maintain complement activity across a broad range of body temperatures, possibly as an adaptation to hibernation (Baker et al. 2013). While these traits have been primarily studied in the context of antiviral immunity, excessive inflammatory responses represent a common pathological pathway between heat stress and active infection.

We conducted a thorough differential diagnosis, acknowledging the limitations posed by the number and condition of available carcasses. Compared to well‐studied species, such as domestic animals, fewer baseline data and diagnostic tools are available for assessing the role of microorganisms and chemicals in similar die‐offs (O'Shea et al. 2016). The advanced decomposition of carcasses led to secondary bacterial overgrowth, preventing the isolation of potential pathogens. However, gross pathology and histological analyses did not indicate active bacterial or parasitic infections. Broad‐spectrum molecular tests did not detect viruses from the genera *Lyssavirus* and *Hantavirus* or the families Coronaviridae and Filoviridae. These viral groups were selected based on their known presence in European bats and their high lethality in both bats and other mammals (Kohl et al. 2021). Moreover, within the constraints of post‐mortem alterations, no histological evidence of viral infection was observed. While we cannot entirely exclude the circulation of pathogens within the studied population, our findings do not suggest an ongoing outbreak. Further research should explore alternative causes of mortality, including exposure to toxic contaminants. A previous study in Germany found that *N. noctula* had higher levels of organochlorine pesticides than other bat species examined (Schanzer et al. 2022). However, such analyses remain challenging due to the lack of established protocols and reference standards for this species, complicating data acquisition and interpretation. Moreover, the toxic effects of these chemicals on bats are poorly understood, making it difficult to relate chemical residue levels to potential population impacts (Schanzer et al. 2022). Given the alignment between the dates of body retrieval and sustained temperature increases approaching or exceeding 30°C, along with the results of laboratory analyses, heatstroke is the most plausible explanation for our observations. Correlative evidence also indicates that admissions to wildlife rehabilitation centres in Italy rise following heatwaves, affecting both adults and juveniles of various species, including those traditionally considered thermophilic (Salinas‐Ramos et al. 2023). *Nyctalus noctula* exhibits higher heat tolerance than other species, withstanding ambient temperatures of up to 48°C (Czenze et al. 2024). However, this tolerance may be linked to the relatively high body mass of adult *N. noctula* among European bat species and may not extend to lactating individuals (Czenze et al. 2024). Additionally, we did not monitor ambient temperatures inside roosts, which are likely to be significantly higher than surrounding environmental measurements due to heat accumulation (Martin Bideguren et al. 2019; Crawford and O'Keefe 2021; Czenze et al. 2022b; Lourenço and Palmeirim 2004). Like artificial bat boxes, natural tree roosts at the forest periphery are vulnerable to overheating during heatwaves, exacerbating risks for bats, particularly newborns with limited thermoregulatory capacity. When heatwaves occur, these roosts can become ecological traps (Salinas‐Ramos et al. 2023; Tanalgo et al. 2025). In the future, using data loggers will enable better monitoring of bat behaviour in response to roost temperatures. Although our sample size was limited, it is noteworthy that only *N. noctula* were found dead, despite both *N. noctula* and *N. lasiopterus* roosting in the same forest patch (Russo et al. 2023). Whether this is due to chance, greater susceptibility to heatwaves in *N. noctula* , or differences in the roosting bats' position in the cavity remains speculative and requires further investigation.

The relationship between roost microclimate and bat thermal physiology (Czenze et al. 2022a) suggests species‐specific differences, with bats occupying hotter roosts potentially developing greater heat tolerance and evaporative cooling capacities. For example, *Hypsugo savii* , a small vespertilionid bat often roosting in highly sun‐exposed human‐made roosts, may reach maximum skin temperatures as high as 46.5°C (Ancillotto et al. 2018). However, this adaptation does not negate the threat of extreme temperature fluctuations, particularly in habitats with poor thermal buffering. For instance, heat tolerance in North American bat species can depend on the type of roost used: hoary bats ( *Lasiurus cinereus* ), which roost in foliage, have higher heat tolerance than little brown bats ( *Myotis lucifugus* ) and silver‐haired bats ( *Lasionycteris noctivagans* ), which roost in tree cavities that are more thermally buffered (Noakes et al. 2021). Likewise, noctules use deep tree cavities, so they may be less heat tolerant than species such as barbastelle bats ( *Barbastella barbastellus* ), which frequently roost under loose bark in snags (Russo et al. 2017). Therefore, the impact of heatwaves on forest bats may be species specific and not easily generalisable, especially considering body mass and phylogeny, which can influence these patterns (Noakes et al. 2021).

In a roost selection study conducted in the same area as the present research, which could not distinguish between *N. noctula* and *N. lasiopterus* , bats avoided roosting near the edges of forest fragments (Russo et al. 2023). This suggests that large peripheral areas may have become unsuitable for roosting, with only the core of the forest fragment being preferred. This further supports the view that forest fragmentation significantly threatens forest bats, such as noctules (Popa‐Lisseanu et al. 2009). Our study extends this perspective by highlighting the vulnerability of these bats to heat stress, particularly in isolated roosts. The discovery of dead newborn noctules, likely killed by heatstroke, underscores the additional threat of extreme temperature fluctuations, especially along forest patch edges. If confirmed, the “edge effect” on roosting bats, likely exacerbated by climate change, could be more severe than previously recognized. While small forest patches in agricultural landscapes may be crucial for tree‐dwelling bats, rising temperatures may progressively make woodland edges unsuitable for roosting, potentially acting as ecological traps (Russo, Jones, Polizzi, et al. 2024; Russo, Jones, Martinoli, et al. 2024) by causing the mortality of pups born there. These insights add a new dimension to the ongoing concerns about forest fragmentation, emphasizing the critical role of roost site thermal conditions in the survival of noctules and other forest bats. They also provide a new perspective on how forest fragmentation may threaten bats, warranting further testing in different environmental and geographic contexts to confirm.

This is the first study to document tree‐dwelling bats dying in response to heatwaves in temperate regions. We acknowledge that our study focused on a single site with a limited sample size. The number of bats we recovered likely underestimates the mortality rate, as many additional dead bats may have been overlooked. Their bodies could have remained inside the roost, been scavenged by local carnivores, or been missed in the forest patch, where dead wood and leaf litter are abundant. Nevertheless, our observations suggest this phenomenon may be far more widespread than currently recognised. Urgent research is needed to assess the full extent of this threat, explore its potential links to land use change and fragmentation, and develop strategies to mitigate its impact. Engaging citizen scientists in systematic searches for bat carcasses during and after extreme heat events could improve our understanding of the true extent and geographic spread of this emerging threat.

## Author Contributions

**Danilo Russo:** conceptualization (lead), formal analysis (lead), investigation (lead), methodology (lead), supervision (lead), writing – original draft (lead), writing – review and editing (lead). **Anne Mäenurm:** data curation (lead), investigation (lead), writing – review and editing (equal). **Luca Cistrone:** investigation (equal), methodology (equal), visualization (equal), writing – review and editing (equal). **Adriano Martinoli:** conceptualization (equal), investigation (equal), writing – review and editing (equal). **Greta Foiani:** formal analysis (lead), investigation (equal), methodology (equal), writing – review and editing (equal). **Valentina Giongo:** formal analysis (equal), investigation (equal), writing – review and editing (equal). **Stefania Leopardi:** formal analysis (lead), funding acquisition (lead), investigation (lead), methodology (lead), writing – original draft (equal), writing – review and editing (equal).

## Conflicts of Interest

The authors declare no conflicts of interest.

## Supporting information


**Table S1.** Summary of bat carcasses retrieved during heatwaves in Northeastern Italy (June 2023–2024), encompassing genetic identification, preservation status, and pathological findings. Gross pathology and histopathology assessments were carried out on necropsy‐suitable specimens. Molecular analyses screened for pan‐viral presence, including *Lyssavirus*, *Hantavirus*, Filoviridae, and Coronaviridae, with all tested samples yielding negative results.

## Data Availability

All data supporting the findings of this study are explicitly described in the main text. No additional datasets were generated or analysed beyond those detailed in the manuscript.
