# Supplementary material for: Climate Change‐Driven Heatwaves Pose Lethal Risks to Newborn Forest Bats
Source: Ecol Evol. 2025 May 13;15(5):e71350. doi: 10.1002/ece3.71350 (PMC12074898; doi:10.1002/ece3.71350)
Supplement: Supplementary file 1 — Table S1. Summary of bat carcasses retrieved during heatwaves in Northeastern Italy (June 2023–2024), encompassing genetic identification, preservation status, and pathological findings. Gross pathology and histopathology assessments were carried out on necropsy‐suitable specimens. Molecular analyses screened for pan‐viral presence, including Lyssavirus, Hantavirus, Filoviridae, and Coronaviridae, with all tested samples yielding negative results. [file ECE3-15-e71350-s001.docx]

**Supplementary Table 1.** Summary of bat carcasses retrieved during heatwaves in Northeastern Italy (June 2023–2024), encompassing genetic identification, preservation status, and pathological findings. Gross pathology and histopathology assessments were carried out on necropsy-suitable specimens. Molecular analyses screened for pan-viral presence, including Lyssavirus, Hantavirus, Filoviridae, and Coronaviridae, with all tested samples yielding negative results.

| ID animal | Date of finding | ID tree | *Genetic* ID | Status of preservation | Gross pathology | Histopathology | Molecular biology pan-virus | | | |
| --- | --- | --- | --- | --- | --- | --- | --- | --- | --- | --- |
|  |  |  |  |  |  |  | Lyssavirus | Hantavirus | Filoviridae | Coronaviridae |
| 1 | 24/06/2023 | 1 | *N. noctula* | mummified |  |  | negative |  |  |  |
| 2 | 19/06/2024 | 1 | *N. noctula* | highly deteriorated |  |  | negative |  |  |  |
| 3 | 19/06/2024 | 2 | *N. noctula* | suitable for necropsy | highly decomposed liver and lungs (grey margins) | Lung: accumulation of polymorphonuclear and mononuclear intravascular cells, sparse activated macrophages in alveoli. Other organs: moderate to severe autolytic changes | negative | negative | negative | negative |
| 4 | 20/06/2024 | 1 | not suitable | suitable for necropsy | pallid heart and lungs | Severe autolytic changes | unsuitable | negative | negative | negative |
| 5 | 20/06/2024 | 1 | *N. noctula* | suitable for necropsy | anemic, highly deteriorated | Liver: accumulation of polymorphonuclear and mononuclear intravascular cells. Other organs: moderate to severe autolytic changes | negative | negative | negative | negative |
| 6 | 21/06/2024 | 6 | *N. noctula* | skeletonised |  |  |  |  |  |  |
| 7 | 21/06/2024 | 6 | not suitable | highly deteriorated |  |  | unsuitable |  |  |  |
| 8 | 21/06/2024 | 6 | *N. noctula* | suitable for necropsy | non-ossified skull | Severe autolytic changes | negative | negative | negative | negative |
| 9 | 21/06/2024 | 6 | *N. noctula* | suitable for necropsy | liquid in the skull and thorax, congested lungs with pallid areas. Stomach full of milk | Lung: accumulation of polymorphonuclear and mononuclear intravascular cells, sparse activated macrophages in the alveoli. Heart: severe autolytic changes, increased interstitial cellularity. Other organs: severe autolytic changes. | unsuitable | negative | negative | negative |
| 10 | 22/06/2024 | 6 | not suitable | skeletonised |  |  |  |  |  |  |
| 11 | 22/06/2024 | 6 | not suitable | mummified |  |  |  |  |  |  |
| 12 | 22/06/2024 | 6 | not suitable | highly deteriorated |  |  | negative |  |  |  |
| 13 | 24/06/2024 | 1 | *N. noctula* | suitable for necropsy | congested skull, yellow margins of lungs. Feces in the rectum. | Liver: accumulation of polymorphonuclear and mononuclear intravascular cells. Lung: accumulation of polymorphonuclear and mononuclear intravascular cells, sparse activated macrophages in alveoli. Other organs: moderate to severe autolytic changes | negative | negative | negative | negative |
| 14 | 26/06/2024 | 6 | not suitable | mummified |  |  |  |  |  |  |
| 15 | 26/06/2024 | 6 | not suitable | mummified |  |  |  |  |  |  |
| 16 | 26/06/2024 | 6 | not suitable | mummified |  |  |  |  |  |  |
| 17 | 26/06/2024 | 6 | not suitable | skeletonised |  |  |  |  |  |  |
